# Prevalence of the Genus *Propionibacterium* in Primary and Persistent Endodontic Lesions: A Systematic Review

**DOI:** 10.3390/jcm9030739

**Published:** 2020-03-09

**Authors:** Mario Dioguardi, Mario Alovisi, Vito Crincoli, Riccardo Aiuto, Giancarlo Malagnino, Cristian Quarta, Enrica Laneve, Diego Sovereto, Lucio Lo Russo, Giuseppe Troiano, Lorenzo Lo Muzio

**Affiliations:** 1 Department of Clinical and Experimental Medicine, University of Foggia, Via Rovelli 50, 71122 Foggia, Italy; giancarlomalagnino@gmail.com (G.M.); cristian_quarta.549474@unifg.it (C.Q.); enrica.laneve@unifg.it (E.L.); diego_sovereto.546709@unifg.it (D.S.); lucio.lorusso@unifg.it (L.L.R.); giuseppe.troiano@unifg.it (G.T.); lorenzo.lomuzio@unifg.it (L.L.M.); 2 Department of Surgical Sciences, Dental School, University of Turin, 10126 Turin, Italy; mario.alovisi@unito.it; 3 Department of Basic Medical Sciences, Neurosciences and Sensory Organs, Division of Complex Operating Unit of Dentistry, “Aldo Moro” University of Bari, Piazza G. Cesare 11, 70124 Bari, Italy; vito.crincoli@uniba.it; 4 Department of Biomedical, Surgical, and Dental Science, University of Milan, 20122 Milan, Italy; Riccardo.Aiuto@unimi.it

**Keywords:** *Propionibacterium*, apical periodontitis, endodontic failure, primary endodontic infection, secondary endodontic infection

## Abstract

*Propionibacterium* are anaerobic/aero-tolerant rod Gram-positive bacteria, and numerous studies are associated with primary and secondary endodontic infections. The data in the literature on the prevalence of *Propionibacterium* are conflicting, and there are studies that report conflicting data on the prevalence in primary and secondary endodontic infections. This review aims to clarify the prevalence of bacteria of the genus *Propionibacterium* in endodontic lesions. The present systematic review work was performed on the basis of the Prisma protocol. A search was carried out on the PubMed and Scopus databases with the use of keywords. The research produced 410 records, which, after the elimination of the overlaps and the application of the inclusion and exclusion criteria, led to a number of 36 included articles divided by the three outcomes. The first outcome concerns prevalence of bacteria of the genus *Propionibacterium* in primary and secondary endodontic lesions. The secondary outcome, differences in the prevalence of bacteria of the genus Propionibacterium between primary endodontic infections and secondary endodontic infections. The tertiary outcome, differences in the prevalence of *Propionibacterium Acnes* compared to *Propionibacterium propionicum* in endodontic infections. The results of the meta-analysis show that the genus *Propionibacterium* bacteria are more prevalent in secondary endodontic infections and that *P. acnes* has a higher prevalence than *P. propionicum*.

## 1. Introduction

The bacteria involved in primary endodontic lesions are mainly aerobic and facultative anaerobes. In the literature the bacterial associations most frequently associated with primary infections are: *Fusobacterium nucleatum*, *Porphyromonas endodontalis*, *Peptostreptococcus micros*, *Campylobacter rectus* [1]; *Prevotella intermedia*, *P. micros*, and *P. anaerobius* eubacteria [2]; and *eubacteria Prevotella*, and *Peptostreptococcus* [3]. For persistent intraradicular and extraradicular secondary infections, the scientific literature focuses on the role of the enterococci (*enterococcus faecalisis*) of streptococci (Gram-positive optional anaerobic bacteria) while calling into question the role of bacteria of the genus *Actinomyces* and of the genus *Propionibacterium* for persistent infections involving the extraradicular apical surface with the formation of bacterial biofilms. *Propionibacterium* are anaerobic/aerotolerant rod Gram-positive bacteria, and numerous studies are associated with primary and secondary endodontic infections; however, the prevalence data are actually conflicting. In fact, Sundqvist et al., 1989 [4] reports the presence of *Propionibacterium* on 1 in 72 in apical periodontitis, while Niazi et al., 2010 reports data with a prevalence of 18 out of 20 in refractory endodontic lesions and Rocas et al., 2011 [5] 26 on 43 necrotic teeth.

Previous systematic reviews have not investigated the role of *Propionibacterium*. The latest review conducted by Prada et al., 2019 on the role of bacteria in endodontic infections did not perform meta-analysis on the prevalence of *Propionibacterium* [6].

This review aims to clarify the prevalence of bacteria of the genus *Propionibacterium* in endodontic lesions. This knowledge is important given the presence of bacteria of the Order of *Actinomycetes* in persistent endodontic infections refractory to endodontic treatments [7].

## 2. Materials and Methods

The following systematic review was conducted based on the indications of the Prisma protocol.

The study was constructed on the population, intervention, control, and outcome (PICO) questions: population (patients with teeth with primary and secondary endodontic infections), intervention (bacteria of the genus *Propionibacterium*), control (patients with teeth that have no *Propionibacterium* infections), and outcome (prevalence of bacteria of the genus *Propionibacterium* in primary and secondary endodontic infections).

The formulation of the PICO question is as follows: What is the prevalence of bacteria of the genus *Propionibacterium* in primary and secondary endodontic infections (primary outcome)? Other questions were also raised: if there is a greater prevalence of *Propionibacterium* in secondary endodontic infections than primary infections (secondary questions) and which among the species of the genus *Propionibacterium* (*P. acnes* and *P. propionicum*) has the greatest prevalence in endodontic lesions (tertiary questions)?

After an initial selection phase of the records identified in the databases, the potentially eligible articles are qualitatively evaluated in order to investigate the role of bacteria in endodontic infections and in apical periodontitis, with particular attention to the role of *Propionibacterium* in endodontic infections.

### 2.1. Eligibility Criteria

The works taken into consideration are clinical studies concerning the role of bacteria in endodontic bacterial infections. In particular, all studies that have investigated the presence of microorganisms within the dental elements subject to endodontic treatment or retreatment conducted in recent years and published with abstracts in English have been considered potentially eligible.

It was decided to choose articles of the last 40 years, because more and more new bacterial species have been identified since 1980 [8], and moreover, the way of classifying and dividing them in the various families has changed. [9] Moreover, the identification methods of bacteria have improved with the introduction of PCR.

The potentially eligible articles were finally subjected to a full-text analysis to verify use for a qualitative analysis and quantitative analysis.

The inclusion and exclusion criteria applied in the full-text analysis are the following:

- Include all those studies that identified the presence of bacteria of the genus *Propionibacterium* in the dental elements subjected to endodontic treatment or retreatment or in the teeth subjected to apicectomy or extraction following endodontic failure.
- The exclusion criteria are: to exclude all those studies that do not report the prevalence data of the bacteria of the genus *Propionibacterium* in the primary and secondary lesions of the dental elements, to exclude all studies reporting a number of teeth examined below 20 for excessive risk of bias, and those not written in English and were published before 1980.

### 2.2. Research Methodology

Studies have been identified through bibliographic research on electronic databases.

The literature search was conducted on the search engines “PubMed” and “Scopus”. The search on the providers was conducted between 07.12.2019 and 22.12.2019, and the last search for a partial update of the literature was conducted on 26/12/2019.

The following search terms were used on PubMed and Scopus: “Propionibacterium” AND “endodontic” OR “apical parodontitis” (PubMed 67), “persistent intraradicular infection” OR “primary endodontic infection” (PubMed 36), “endodontic failure” OR “endodontic microbiologic” (PubMed 201), persistent intraradicular infection (Scopus 23), “persistent extraradicular infection” (Scopus 18), and “Propionibacterium” AND “endodontic” (Scopus 65) (Table 1).

### 2.3. Screening Methodology

The keywords to be searched and their combinations were decided before the identification phase of the records in common agreement between the two reviewers (with the task of selecting potentially eligibile articles). The records obtained were subsequently examined by two independent reviewers (M.D. and C.Q.), and a third reviewer (G.T.) acted as a decision-maker in situations of doubt.

The screening included the analysis of the title and the abstract and, in doubtful cases, of text analysis to eliminate the records not related to the topics of the review. The articles obtained were subjected to full-text analysis by the two reviewers (80 articles), from which the ones eligible for the qualitative analysis and inclusion in the meta-analysis for the three outcomes were identified. The results sought by the two reviewers were:

(1) Primary outcome, the prevalence of bacteria of the genus *Propionibacterium* in primary and secondary endodontic lesions;
(2) Secondary outcome, differences in the prevalence of bacteria of the genus *Propionibacterium* between primary endodontic infections and secondary endodontic infections;
(3) Tertiary outcome, differences in the prevalence of *Propionibacterium Acnes* compared to *Propionibacterium propionicum* in endodontic infections.

The K agreement between the two screening reviewers was 0.8584 (Table 2) [10]. The K agreement was based on the formulas of the Cochrane Handbook for Systematic Reviews [11].

**Figure 1:**
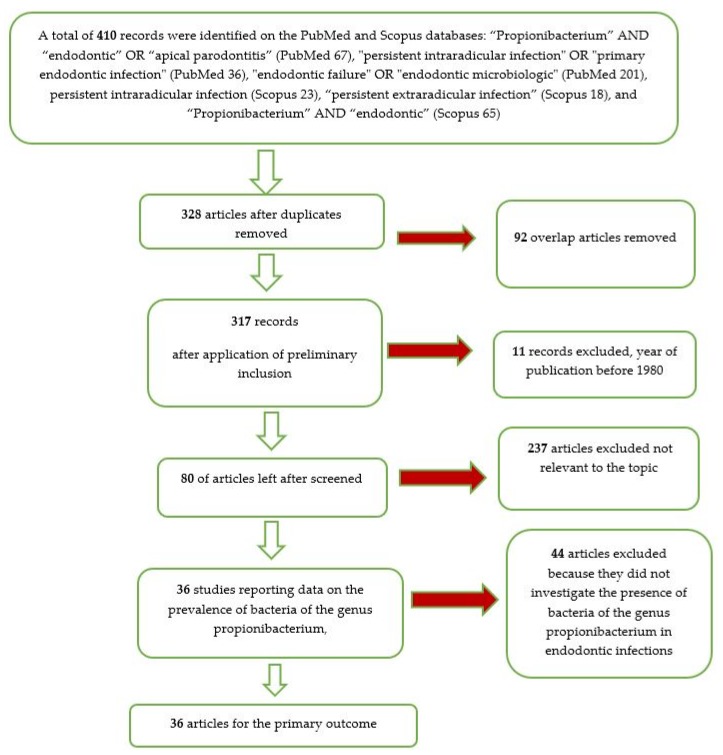
Flow chart of the different phases of the systematic review.

The entire selection and screening procedures are described in a flow chart (Figure 1).

The Newcastle–Ottawa Scale for case control studies was used to assess the risk of bias in the included studies in primary, secondary, and tertiary outcomes [12]. The cumulative meta-analysis for the first outcome was performed using the software Open Meta-Analyst version 10; the quantitative analysis for the secondary and tertiary outcomes was performed with the Rev Manager software 5.3 (Cochrane Collaboration, Copenhagen, Denmark). The data have been processed following the indications of the Cochrane Handbook for Systematic Reviews Chapters 7.4, 7.5, and 7.6 [11].

## 3. Results

From the searches in the PubMed and Scopus databases, 410 records were identified. With the use of the end-note software, the overlaps were removed, obtaining 328 records. After the elimination of the articles prior to 1980, we reach a number of records of 317. With the application of the eligibility criteria (all the studies that investigated the presence of bacteria in endodontic infection), we reach a number of 80 articles.

Applying the inclusion and exclusion criteria, we included 36 articles in the meta-analysis.

- Thirty-six articles for the primary outcome: all studies reporting data on the prevalence of bacteria of the genus *Propionibacterium*, further divided into two subgroups A and B. A—prevalence of the genus *Propionibacterium* in primary endodontic infections in untreated canals, pulp necrosis, and pulpits (21 articles) and B—prevalence of the genus *Propionibacterium* in secondary endodontic infections in treated channels, in cases of endodontic failure and in cases of endodontic retreatment (20 articles);
- Seven articles for the secondary outcome: all studies reporting data differences in the prevalence of bacteria of the genus *Propionibacterium* between primary endodontic infections and secondary endodontic infections; and
- Fourteen articles for the tertiary outcome: all studies reporting data differences in the prevalence of *Propionibacterium Acnes* compared to *Propionibacterium propionicum* in endodontic infections.

### 3.1. Study Characteristics and Data Extraction

The included studies for the quantitative analysis were:First Outcome: Pourhajibagher et al., 2018 [13]; Grgurevic et al., 2017 [14]; Lysakowska et al., 2016 [15]; Tennert et al., 2014 [16]; Halbauer et al., 2013 [17]; Signoretti et al., 2013 [18]; Anderson et al., 2013 [19]; Rocas et al., 2012 [20]; Rocas et al., 2011 [5]; Chugal et al., 2011 [21]; Ledezma-Rasillo et al., 2010 [22]; Mindere et al., 2010 [23]; Niazi et al., 2010 [24]; Fujii et al., 2009 [25]; Vianna et al., 2007 [26]; Chu et al., 2005 [27]; Chavez de Paz et al., 2005 [28]; Gomes et al., 2004 [29]; Chavez de Paz et al. [30]; Siqueira et al., 2004 [31]; Hommez et al., 2004 [32]; Pinheiro et al., 2003 [33]; Siqueira et al., 2003 [34]; Sunde et al., 2002 [35]; Peters et al., 2002 [36]; Rolph et al., 2001 [37]; Sundqvist et al., 1998 [38]; Molander et al., 1998 [39]; Vigil et al., 1997 [40]; Sjogren et al., 1997 [41]; Gomes et al., 1996 [42]; Brauner et al., 1995 [43]; Debelian et al., 1995 [44]; Sundqvist et al., 1992 [45]; Fukushima et al., 1990 [46]; and Sundqvist et al., 1998 [4].Second Outcome: Lysakowska et al., 2016 [15]; Signoretti et al., 2013 [18]; Chugal et al., 2011 [21]; Gomes et al., 2004 [29]; Hommez et al., 2004 [32]; Rolph et al., 2001 [37]; and Siqueira et al., 2003 [34].Third Outcome: Lysakowska et al., 2016 [15]; Tennert et al., 2014 [16]; Halbauer et al., 2013 [17]; Ledezma-Rasillo et al., 2010 [22]; Niazi et al., 2010 [24]; Vianna et al., 2007 [26]; Chu et al., 2005 [27]; Chavez de Paz et al., 2005 [28]; Chavez de Paz et al., 2004 [30]; Pinheiro et al., 2003 [33]; Siqueira et al., 2003 [34]; Sunde et al., 2002 [35]; Peters et al., 2002 [36]; Sundqvist et al., 1998 [38]; Sjogren et al., 1997 [41]; and Debelian et al., 1995 [44].

The extraction of the data and the methods in which they have been reported follow the indications of the Cochrane Handbook for Systematic Reviews of Interventions Chapter 7 (Selection of Studies and Data Collection), specifically from pages 152 to 182.

The extracted data included the magazine (author, data, and journal); the bacterium species of the genus *Propionibacterium* investigated (genus, species, and number of dental elements with the presence of the bacterium); the number of samples examined; types of samples (necrotic or vital tooth, endodontic canal, tooth in pulpitis or apical periodontitis, tooth previously treated endodontically, and tooth with failure subject to extraction or endodontic surgery); the number of samples per pathology with the presence of *Propionibacterium*; and the bacterium identification method (PCR or culture). If the data on the prevalence in the single studies were reported only for the individual species of *Propionibacterium* and the overall data was not present or it was not possible to obtain it, the data pertaining to the species was considered for the purpose of the meta-analysis that, in the single study, presented the higher prevalence. If the data was reported as a percentage, the number was calculated through the use of proportions.

The data extracted for the tree outcomes are shown in Table 3, Table 4 and Table 5.

### 3.2. Risk of Bias

The risk of bias was assessed through the Newcastle–Ottawa case control scale, modified for the cumulative meta-analysis. The results are reported in detail in Table 6. For each category, a value of one to three was assigned (one = low and three = high).

Studies presenting a high risk of bias were not included in the meta-analyzes. Articles with high bias risk were excluded from the scale and eliminated during the inclusion phase. Other articles were excluded, because, for the outcomes investigated, they presented the same data and samples. Some studies, although presenting a number of samples greater than or equal to 20, do not report precise data on the exact number of *Propionibacterium* in relation to the dental elements subject to endodontic lesions (for example, Francisco et al., 2018 [47,48]). Other studies, such as that conducted by Mussano et al., 2018 [48], even if starting from a number of patients equal to 121 with acute apical periodontitis, only 10 biopsy specimens were examined. The bias risk assessment of the 36 articles included was conducted by the first reviewer (M.D.).

**Figure 2:**
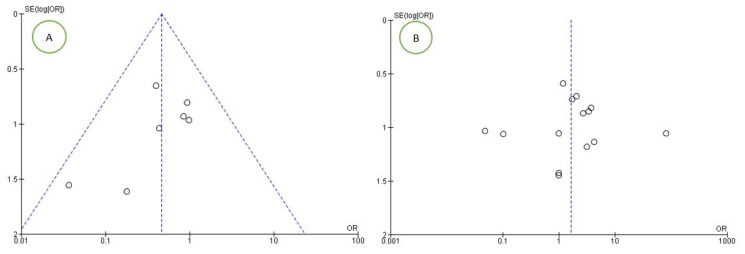
Funnel plots of the evaluation of heterogeneity for the (**A**) second and (**B**) third outcomes.

For the first outcome, the risk of bias between studies was very high and was evidenced by the high heterogeneity between studies (I2 92.78%). For the secondary outcome, the risk of bias between studies was low, as represented by the funnel plot (Figure 2A). For the tertiary outcome, the risk was average, with a heterogeneity between studies of I2 65%, and was assessed with the funnel plot (Figure 2B).

### 3.3. Meta-Analysis

The statistical analysis of the data was performed using the Rev Manager 5.3 software (Copenhagen, 153 Denmark, The Nordic Cochrane Centre, The Nordic Cochrane Collaboration, 2014), and cumulative meta-analysis for the first outcome was performed using the software Open Meta-Analyst version 10. The results were represented by forest plots for each of the outcomes.

**Figure 3:**
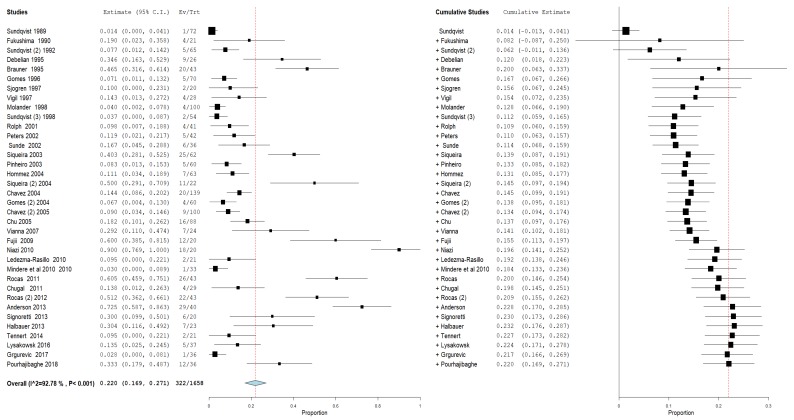
Forest plot of the random effects model of the meta-analysis cumulative of the primary outcome.

For the first outcome, prevalence of bacteria of the genus *Propionibacterium* in primary and secondary endodontic lesions, the heterogeneity was very high, with I^2^ equal to 92.78%. For this reason, a random effects model was used. The cumulative meta-analysis presents an Overvall (I^2^= 92.78%; *p*-value < 0.001) of 0.202 (0.169 and 0.279) with a ratio between events and samples examined equal to 322\1658 (Figure 3).

**Figure 4:**
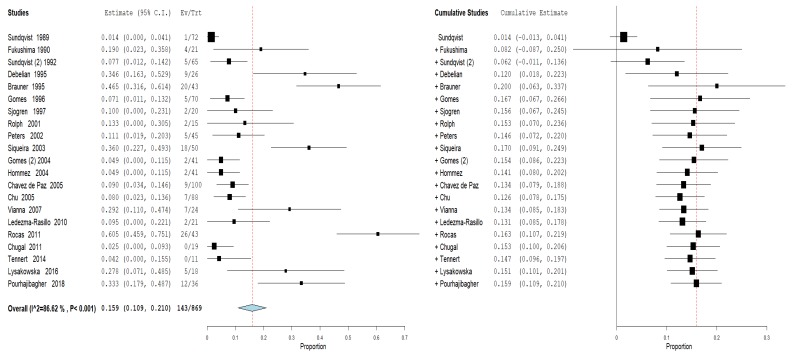
Forest plot of the random effects model of the meta-analysis cumulative of the primary outcome subgroup A.

**Figure 5:**
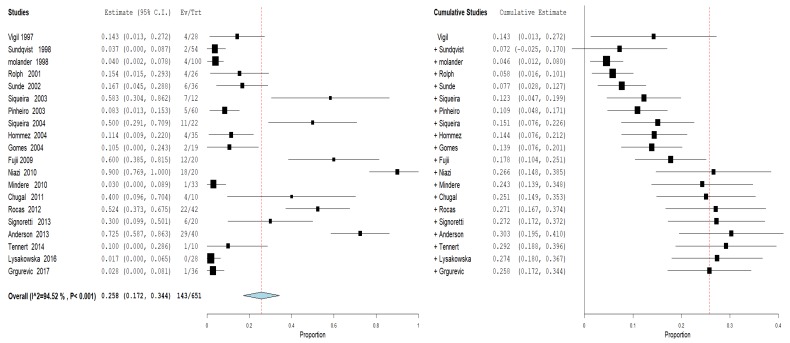
Forest plot of the random effects model of the meta-analysis cumulative of the primary outcome subgroup B.

Furthermore, an analysis of the subgroups was performed: subgroups A—prevalence of the genus *Propionibacterium* in primary endodontic infections in untreated canals, pulp necrosis, and pulpits (Figure 4). For subgroup A, the heterogeneity is very high, with I^2^ equal to 86.62%. For this reason, a random effects model was used. The cumulative meta-analysis presents an Overvall (I^2^ = 86.62%; *p*-value < 0.001) of 0.159 (0.109 and 0.210) with a ratio between events and samples examined equal to 143\869. B—prevalence of the genus *Propionibacterium* in secondary endodontic infections in treated channels in cases of endodontic failure and in cases of endodontic retreatment (Figure 5). For subgroup B, the heterogeneity is very high, with I^2^ equal to 94.52%. For this reason, a random effects model was used. The cumulative meta-analysis presents an Overvall (I^2^ = 94.52%; *p*-value < 0.001) of 0.258 (0.172 and 0.344) with a ratio between events and samples examined equal to 143\651.

For the secondary outcome, differences in the prevalence of bacteria of the genus *Propionibacterium* between primary endodontic infections and secondary endodontic infections, the comparison showed absence of heterogeneity among the studies, with an I^2^ equal to 0%. For this reason, for the second outcome, a fixed effects model was applied. For the second outcome, the forest plot is in favor of the subject group primary endodontic infection in a statistically significant way.

**Figure 6:**
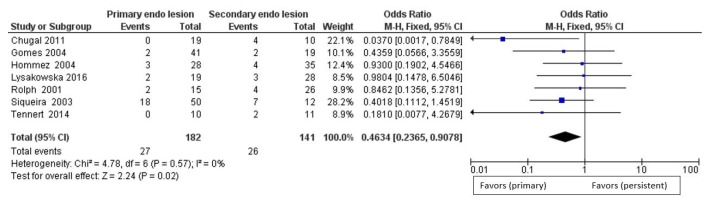
Forest plot of the fixed effects model of the meta-analysis of the secondary outcome.

All studies show data in favor of a higher prevalence of *Propionibacterium* in secondary endodontic infections. The forest plot shows the rhombus that does not intersect the noneffect line, moved towards primary endodontic infections having fewer events (presence of *Propionibacterium*) in relation to the population (teeth with infection) compared to the group of secondary endodontic infections (Figure 6).

**Figure 7:**
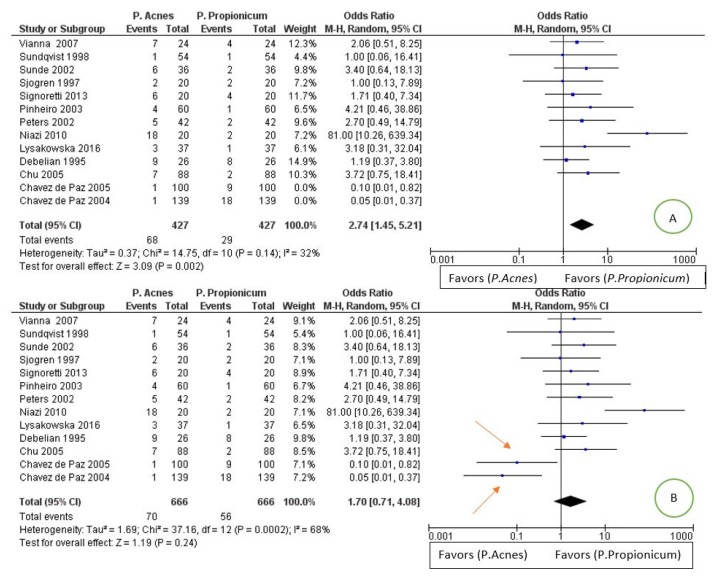
Image A: forest plot of the random effects model of the meta-analysis of the tertiary outcome; the arrows indicate the main sources of heterogeneity. Image B: forest plot with the exclusion of heterogeneity sources.

For the tertiary outcome: difference in the prevalence of *Propionibacterium Acnes* compared to *Propionibacterium propionicum* in endodontic infections. The comparison showed high heterogeneity between the studies, with an I^2^ of 68%; a random effects model was applied. Through an exploration of the sources of heterogeneity, we noted that, from excluding the studies of Chavez de Paz et al., 2004 [30] and Chavez de Paz et al., 2005 [28], heterogeneity decreases from 68% to 32% (as shown in Figure 7).

The forest plot is in favor of the *Propionibacterium propionicum* group, having a smaller number of events in relation to the population in proportions less than the group of the *Propionibacterium acnes*.

## 4. Discussion

The endodontic treatment is a reasonably predictable procedure with success rates between 86% and 98%. The success or failure of this treatment is also assessed by clinical signs and symptoms, as well as radiological results of the treated tooth [49].

A number of studies have focused on the detection and identification of microorganisms in the root canal of root-filled teeth [33,50,51], and the persistence of microorganisms in the apical part of the root canal was recognized as the main cause of failure of endodontic treatment, even after performing lege artis of endodontic procedures [52]. This can occur due to the inability of endodontic instruments and irrigants to reach all parts of the canal system and effectively remove microorganisms [53,54]. The root canal microflora between primary endodontic cases and retreatment cases differs [38].

The main problem is that, in most cases, the apico-coronal seal is inadequate; therefore, tissue fluids rich in glycoproteins go into the root canal, providing a substrate for the remaining microorganisms, which can proliferate and reach a sufficient number to generate or perpetuate a periradicular lesion [55]. On the other hand, there are situations in which the sealed root canals may be contaminated by the oral cavity: infiltration through temporary or permanent restoration materials, fracture or loss of restoration, fracture of the tooth structure, recurring caries that expose the root-filling material, or delay in the application of the final restoration material. In these circumstances, if a root filling does not prevent saliva from entering, microorganisms can invade and recolonize the canal system [56].

Bacterial survival is closely related to their ability to adapt to hostile environments, and biofilm formation is considered an effective survival strategy and a common cause of persistent infection [57].

To survive in a sealed channel, microorganisms must withstand intracanal disinfection measures and must adapt to an environment with poor nutrient availability. Therefore, only the species that have these abilities may be involved in endodontic failure. Furthermore, bacteria located in areas such as apical deltas, lateral canals, irregularities, and dentinal tubules can often escape endodontic disinfection procedures, and it is likely that the supply of bacterial nutrients remains unchanged after treatment [58].

The microorganisms that reach the environment beyond the foramen of the root canal are recognized by the immune system, initiating a local inflammatory response—a series of events with the aim of eliminating the infection and providing conditions for restoring the balance of the guest [59].

However, many microorganisms could survive due to their ability to bypass, respond to, or resist host defense mechanisms, colonizing the external root surface and forming a biofilm [60]. Microbial species associated with bacterial complexes organized in a biofilm possess characteristics that differ from their planktonic forms, such as greater diversity and metabolic efficiency, resistance to phagocytic cells, antimicrobial agents and environmental stresses, and increased pathogenicity [61,62].

Bacterial colonization of root canal spaces has been demonstrated as the main etiological factor of apical periodontitis [63,64]. In most cases, it is impossible to distinguish between periapical granulomas and radicular cysts without resorting to a biopsy [65]. Radicular cysts are believed to form from the proliferation of Malassez epithelial cell remains in inflamed periradicular tissues [66]; their reported incidences among periapical lesions varies from 6% to 55% [67].

In two paradigmatic studies of Ricucci and Siqueira [64,68], bacterial biofilm varied, and no single model for endodontic infections was identified. Bacteria could change the severity and the prognosis of apical periodontitis, and yet, surprisingly, little information is available in the scientific literature comparing the microbiota within periapical granulomas and radicular cysts. The application of high throughput amplicon target sequencing (HTS) to study microbial ecology has been seen over the past two years to estimate microbial diversity in different ecosystems using the 16S rRNA gene as a target [69].

Microbial identification by molecular methods has been widely used in microbiological research applied to dentistry [70,71,72,73,74,75]. Culture-independent molecular biology methods have advantages over bacterial identification procedures based on phenotypic features, such as greater sensitivity and specificity and ability to identify noncultivable bacteria [70,73,76], which generates more reliable results regarding microbial content.

Endodontic surgical treatment is recommended for teeth with long-lasting apical lesions that persist even after careful conventional endodontic retreatment [77,78]. The main objective of apical surgery is to remove the etiological agent, which is normally associated with extraradicular biofilm on long-lasting apical lesions [79,80], periapical actinomycosis [81], foreign body reactions triggered by extruded endodontic materials [82], accumulation of endogenous cholesterol crystals in apical tissues [83], or unresolved cystic lesions [67].

The bacteria that are most frequently found in the root canals of teeth with post-treatment endodontic disease when using culture techniques are predominantly Gram-positive, including cocci (e.g., *Enterococcus* spp. and *Streptococcus* spp.) and rods (e.g., *Actinomyces* and *Propionibacterium*) [33,38,39,84].

Although several bacterial species from the oral cavity have been found in the infected root canals, there is a limited set of species that are most frequently present in some types of endodontic infections and, therefore, are recognized as the main group of endodontic [85]. Among this set, there are some species of the genus *Propionibacterium* that have often been isolated from both intraradicular and extraradicular infections [38,86,87].

The species of *Propionibacterium* detected in the endodontic infections described in the literature are *P. acnes*, *P. propionicum* (the *P. propionicum* and *P. propionicus* are the same bacterium with different names), *P. acidipropionici*, *P. granulosum*, *P. avidium*, and *P. acidifaciens*. From the qualitative analysis of the articles, it appears that the *Propionibacterium* most associated with the presence of endodontic infections is P. acnes, followed by *P. propionicum* and *P. granulosam*.

The results of our meta-analysis for the first outcome shows how the presence of *Propionibacterium* in endodontic infections has a prevalence of 0.202 (0.169 and 0.279) (relationship between teeth with the presence of *Propionibacterium* and teeth with infections) that from the analysis of the subgroups increased from subgroup A (primary endodontic infections) 0.159 to B (secondary endodontic infections) 0.258. It could be assumed that the bacteria of the genus *Propionibacterium* are more present in secondary infections, but further data and analyses are necessary.

In fact, for the second outcome, the data provide us with significant information, thanks to the direct comparison (within the studies) between primary and secondary lesions. *Propionibacterium* is more present in endodontic secondary lesions significantly, confirming in part the data of the analysis of subgroups A and B. The studies by Lysakowska et al., 2016 [15]; Signoretti et al., 2013 [18]; Gomes et al., 2004 [29]; Hommez et al., 2004 [32]; Rolph et al., 2001 [37]; and Siqueira et al., 2003 [34] all report data with a lower presence of *Propionibacterium* in primary endodontic infections but with confidence intervals that intersect the noneffect line, with the exception of Chugal et al., 2011 [21]. The absence of heterogeneity between the studies and the set of studies instead shows a statistically significant result in favor of a lower presence of *Propionibacterium* in primary endodontic infections.

The meta-analysis of the tertiary outcome reports data that are in favor of a higher prevalence of *P. acnes* than *P. propionicum* in a statistically not significant way but with a high heterogeneity of I2 68%. Through a search for the sources of heterogeneity, and excluding the articles of Chavez de Paz et al., 2004 [30] and Chavez de Paz et al., 2005 [28], the heterogeneity is lowered to 32%, and the data become significant and are in favor for fewer lesions/infections in which the *P. propionicum* is present.

All the studies except for the Chavez de Paz et al., 2004 [30] and Chavez de Paz et al., 2005 [28] and Niazi et al., 2010 [57] report confidence intervals that intersect the line of noneffect. In fovor studies for a greater prevalence of *P. propionicum* are Chavez de Paz et al., 2004 [63] and Chavez de Paz et al., 2005 [61], while the studies of Sundqvist et al., 1998 [38] and Sjogren et al., 1997 report identical results for the two groups. The remaining studies are in favor of a greater presence of *P. acnes* in endodontic infections.

## 5. Conclusions

In conclusion, we can say that *Propionibacterium* is definitely present in endodontic infections with a higher prevalence for secondary endodontic lesions and that the *Propionibacterium* that has the highest prevalence is *P. acnes*.

## Figures and Tables

**Table 1:** Complete overview of the search methodology. Records identified by databases: 410 and records selected for quantitative analysis: 36.

Database-Provider	Keywords	Search Details	Number of Records	Articles after Removal of Overlap Articles	Number of Records After Restriction by Year of Publication (last 40 Years)	Numbers of Articles That Have Investigated the Role of Bacteria in Endodontic Infections	Number of Articles Reporting Data on the Presence of Bacteria of the Genus *Propionibacterium* in Teeth Presenting Endodontic Infections Subject to Surgery and Endodontic Treatment\Retreatment
PubMed	Propionibacterium AND endodontic OR apical parodontitis	(“Propionibacterium” (MeSH Terms) OR “Propionibacterium” (All Fields)) AND endodontic (All Fields) OR (apical (All Fields) AND parodontitis (All Fields))	67				
PubMed	“persistent intraradicular infection” OR “primary endodontic infection”	“persistent intraradicular infection” (All Fields) OR “primary endodontic infection” (All Fields)	36				
PubMed	“endodontic failure” OR “endodontic microbiologic”	“endodontic failure” (All Fields) OR (endodontic (All Fields) AND microbiologic (All Fields))	201				
Scopus	persistent intraradicular infection	TITLE-ABS-KEY (persistent AND intraradicular AND infection)	23				
Scopus	persistent extraradicular infection	TITLE-ABS-KEY (persistent AND extravascular AND infection)	18				
Scopus	Propionibacterium AND endodontic	TITLE-ABS-KEY (Propionibacterium AND endodontic)	68				
Total			410	328	317	80	36

**Table 2:** K agreement calculation: Po = 0.925 (proportion of agreement) and Pe = 0.4701 (agreement expected), and so K agreement = 0.8584. No agreement, 0.0–0.20: slight agreement, 0.21–0.40: fair agreement, 0.41–0.60: moderate agreement, 0.61–0.80: substantial agreement, and 0.81–1.00: almost perfect agreement. The K agreement was calculated from the 36 articles to include 15 articles with the application of the inclusion and exclusion criteria.

/	/	Reviewer 2	Reviewer 2	Reviewer 2	
		Include	Exclude	Unsure	Total
**Reviewer 1**	**Include**	36	2	1	39
**Reviewer 1**	**Exclude**	1	37	1	39
**Reviewer 1**	**Unsure**	1	0	1	2
	**Total**	38	39	3	80

**Table 3:** Primary outcome (the data regarding the prevalence of bacteria of the genus *Propionibacterium* in the various studies are reported).

Author, Date, Journal	Species and Genus and Number of Teeth on which *Propionibacterium* is Detected		Dental Pathology, Number of Teeth with *Propionibacterium*			Identification Method
			Pathology	Event	Total	
[13] Pourhajibagher et al., 2018 Photodiagnosis and Photodynamic Therapy	*P. acnes*	12	primary endodontic infections	12\36	36	culture
[14] Grgurevic et al., 2017 Acta Stomatol Croat	*P. propionicum*	1	persistent apical periodontitis	1\36	36	PCR
[15] Lysakowska et al., 2016 International Endodontic journal	*P. acnes*	3\19	primary endodontic infections	5\19	37	culture
		0\28				
	*P. propionicum*	1\19				
		0\28	secondary treatment	0\28		
	*P. Granulosum*	1\19				
		0\28				
[16] Tennert et al., 2014 Journal of Endodontics	*P. acidifaciens*	0\11	primary infection	0\11	21	PCR
		1\10				
	*P. propionicus*	0\11	Secondary/persistent infection	1\10		
		1\10				
[17] Halbauer et al., 2013 Coll Antropol	*P. acnes*	7\23	chronical apical periodontitis (*n* = 17 untreated teeth)	7\23	23	culture
			chronical apical periodontitis (*n* = 6 retreatments)			
[18] Signoretti et al., 2013 Journal of Endodontics	*P. acnes*	2\13	persistent apical lesions associated with well-performed endodontic retreatment (*n* = 13; cyst *n* = 7 granuloma)	6\20	20	culture
		4\7				
	*P. propionicum*	2\13				
		2\7				
[19] Anderson et al., 2013 Journal of Endodontics	*Propionibacterium spp.*	11\17	endodontic infections associated with root-filled teeth (symptomatic *n* = 17;asymptomatic *n* = 23)	29\40	40	PCR
		18\23				
[20] Rocas et al., 2012 Journal of Clinical Microbiology	*P. acnes*	22\42	persistent/secondary infection of the dental root canal	22\42	42	PCR
	*P. acidifaciens*	6\42				
[5] Rocas et al., 2011 Journal of Endodontics	*P acnes*	26\43	necrotic root canals of teeth with symptomatic (*n* = 13) or asymptomatic (*n* = 21) apical periodontitis and chronic apical abscesses (*n* = 9)	26\43	43	PCR
[21] Chugal et al., 2011 Journal of Endodontics	*Propionibacterium spp.*	0\19	primary infection (19 samples); secondary infection (10 samples)	4\29	29	PCR
		4\10				
[22] Ledezma-Rasillo et al., 2010 The Journal of Clinical Pediatric Dentistry	*P. propionicus*	1\21	primary teeth with necrotic pulps	2\21	21	culture
	*P. acnes*	1\21				
[23] Mindere et al., 2010 Stomatologija	*P. avidum*	1\33	root-filled teeth with apical periodontitis	1\33	33	culture
[24] Niazi et al., 2010 Journal of Endodontics	*P. acnes*	18\20	20 refractory endodontic lesions (9 with abscesses and 11 without abscesses)	18\20	20	PCR
	*P. granulosum*	1\20				
	*P. propionicum*	2\20				
	*P. avidum*	2\20				
	*Propionibacterium spp.*	1\20				
[25] Fujii et al., 2009 Oral Microbiology and Immunology	*P. acidipropionici*	1\20	20 apical periodontitis lesions of obturated teeth	12\20	20	PCR
	*P. acnes*	12\20				
[26] Vianna et al., 2007 Oral Microbiology and Immunology	*P. propionicum*	4\24	necrotic pulp apical periodontitis	7\24	24	culture
	*P. acnes*	7\24				
[27] Chu et al., 2005 Journal of Endodontics	*P. acnes*	7\88	primary endodontic infections with exposed (*n* = 45) and unexposed (*n* = 43) Pulp space	7\88	88	culture
	*P. granulosum*	1\88				
	*P. propionicus*	2\88				
[28] Chavez de Paz et al., 2005 Oral Surgery, Oral Medicine, Oral Pathology, Oral Radiology, and Endodontics	*P. acnes*	1\100	primary endodontic infections	9\100	100	PCR
	*P. propionicum*	9\100				
[29] Gomes et al., 2004 Oral Microbiology and Immunology	*P. acnes*	2\41	41 primary infection	4\60	60	PCR
		2\19	19 endodontic failure			
[30] Chavez de Paz et al., 2004 International Endodontic Journal	*P. acidpropionici*	0\139	139 teeth undergoing root canal treatment	20\139	139	PCR
	*P. acnes*	1\139				
	*P. propionicum*	18\139				
	*Propionibacterium spp.*	1\139				
[31] Siqueira et al., 2004 Oral Surgery, Oral Medicine, Oral Pathology, Oral Radiology, and Endodontics	*P. propionicum*	11\22	22 root-filled teeth with persistent periradicular lesions	11\22	22	PCR
[32] Hommez et al., 2004 International Endodontic Journal	*P. granulosum*	3\28	necrosis 28	7\63	63	PCR
		4\35				
	*P. acnes*	1\28				
		2\35	Filled 35 (retreatment)			
	*Propionibacterium spp.*	2\28				
		2\35				
[33] Pinheiro et al., 2003 International Endodontic Journal	*P. acnes*	4\60	60 root-filled teeth with apical periodontitis	5\60	60	culture
	*P. propionicum*	1\60				
[34] Siqueira et al., 2003 Oral Surgery, Oral Medicine, Oral Pathology, Oral Radiology, and Endodontics	*P. propionicus*	18\50	50 cases of untreated (chronic asymptomatic *n* = 21, acute apical periodontitis *n* = 10, and untreated with abscesses *n* = 19)	25\62	62	PCR
		7\12	12 cases of root-filled teeth			
[35] Sunde et al., 2002 Journal of Endodontics	*P. acnes*	6\36	refractory apical periodontitis	6\36	36	culture
	*P. granulosum*	2\36				
	*P. propionicum*	2\36				
[36] Peters et al., 2002 International Endodontic Journal	*P. acnes*	5\42	42 untreated cases	5\42	42	culture
	*P. propionicum*	2\42				
[37] Rolph et al., 2001 Journal of Clinical Microbiology	*P. acnes*	2\15	untreated cases2\15	4\41	41	PCR
		2\26				
	*P. granulosum*	0\15	refractory cases 4\26			
		2\26				
[38] Sundqvist et al., 1998 Oral Surgery, Oral Medicine, Oral Pathology, Oral Radiology, and Endodontics	*P. acnes*	1\54	54 teeth with failed endodontic treatment	2\54	54	culture
	*P. propionicum*	1\54				
[39] Molander et al., 1998 International Endodontic Journal	*Propionibacterium spp.*	4\100	100 root-filled teeth	4/100	100	culture
[40] Vigil et al., 1997 Journal of Endodontics	*P. acnes*	4\28	28 refractories, endodontic cases requiring surgical intervention	4\28	28	culture
[41] Sjogren et al. 1997 International Endodontic Journal	*P. acnes*	2\20	20 apical periodontitis	2\20	20	culture
	*P. propionicum*	2\20				
[42] Gomes et al., 1996 J Dental	*P. acnes*	5\70	necrotic pulp	5/70	70	culture
[43] Brauner et al., 1995 International Endodontic Journal	*P. acnes*	13\19	apical periodontitis (*n* = 19 root canal and *n* = 24 with periapical granuloma)	20\43	43	culture
		7/24				
[44] Debelian et al., 1995 Endodontics & Dental Traumatology	*P. acnes*	9\26	26 teeth with asymptomatic apical periodontitis	9\26	26	culture
	*P. propionicus*	8\26				
	*P. granulosum*	2\26				
[45] Sundqvist et al., 1992 Oral Microbiology and Immunology	*Propionibacterium spp.*	2\65	65 infected human root canals	5\65	65	culture
	*P. propionicus*	5\65				
[46] Fukushima et al., 1990 Journal of Endodontics	*P. acnes*	4\21	21 untreated cases	4\21	21	culture
	*P. acidipropionici*	3\21				
[4] Sundqvist et al., 1989 Journal of Endodontics	*Propionibacterium spp.*	1\72	apical periodontitis	1\72	72	culture

**Table 4:** Secondary outcome (difference in the prevalence of bacteria of the genus *Propionibacterium* between primary endodontic infections and secondary endodontic infections).

Author, Date, Journal	Species	Primary Endodontic Infections		Secondary/Persistent Infection		Identification Method
		Event	Total	Event	Total	
[15] Lysakowska et al., 2016 International Endodontic Journal	*P. acnes*	0	19	3	28	culture
	*P. propionicum*	1	19	0	28	
	*P. Granulosum*	1	19	0	28	
	*P. Acidifaciens*	\	\	\	\	
	*Propionibacterium spp.*	\	\	\	\	
	*Tot*	2	19	3	28	
[16] Tennert et al., 2014 Journal of Endodontics	*P. acnes*	\	\	\	\	PCR
	*P. propionicum*	0	10	1	11	
	*P. Granulosum*	\	\	\	\	
	*P. Acidifaciens*	0	10	1	11	
	*Propionibacterium spp.*	\	\	\	\	
	*Tot*	0	10	2	11	
[29] Gomes et al., 2004 Oral Microbiology and Immunology	*P. acnes*	2	41	2	19	culture
	*P. propionicum*	\	\	\	\	
	*P. Granulosum*	\	\	\	\	
	*P. Acidifaciens*	\	\	\	\	
	*Propionibacterium spp.*	\	\	\	\	
	*Tot*	2	41	2	19	
[32] Hommez et al., 2004 International Endodontic Journal	*P. acnes*	1	28	2	35	PCR
	*P. propionicum*	\	\	\	\	
	*P. Granulosum*	3	28	4	35	
	*P. Acidifaciens*	\	\	\	\	
	*Propionibacterium spp.*	2	28	2	35	
	*Tot*	3	28	4	35	
[34] Siqueira et al., 2003 Oral Surgery, Oral Medicine, Oral Pathology, Oral Radiology, and Endodontics	*P. acnes*	\	\	\	\	PCR
	*P. propionicum*	18	50	7	12	
	*P. Granulosum*	\	\	\	\	
	*P. Acidifaciens*	\			\	
	*Propionibacterium spp.*	\	\	\	\	
	*Tot*	18	50	7	12	
[21] Chugal et al., 2011 Journal of Endodontics	*P. acnes*	\	\	\	\	PCR
	*P. propionicum*	\	\	\	\	
	*P. Granulosum*	\	\	\	\	
	*P. Acidifaciens*			\	\	
	*Propionibacterium spp.*	0	19	4	10	
	*Tot*	0	19	4	10	
[37] Rolph et al., 2001 Journal of Clinical Microbiology	*P. acnes*	2	15	2	26	PCR
	*P. propionicum*	\	\	\	\	
	*P. Granulosum*	0	15	2	26	
	*P. Acidifaciens*	\	\	\	\	
	*Propionibacterium spp.*	\	\	\	\	
	*Tot*	2	15	4	26	

**Table 5:** Tertiary outcome difference in the prevalence of *Propionibacterium Acnes* compared to *Propionibacterium propionicum* in endodontic infections.

Author, Date, Journal	Type of Infection	*Propionibacterium acnes*		*Propionibacterium propionicum*		Identification method
		Event	Tot	Event	Tot	
[15] Lysakowska et al., 2016 International Endodontic Journal	primary endodontic infections	3	19	1	19	culture
	Secondary/persistent infection	0	28	0	28	
	total	3	37	1	37	
[18] Signoretti et al., 2013 Journal of Endodontics	primary endodontic infections	\	\	\	\	culture
	Secondary/persistent infection	6	20	4	20	
	total	6	20	4	20	
[24] Niazi et al., 2010 Journal of Endodontics	primary endodontic infections	\	\	\	\	PCR
	Secondary/persistent infection	18	20	2	20	
	total	18	20	2	20	
[22] Ledezma-Rasillo, 2010 The Journal of Clinical Pediatric Dentistry	primary endodontic infections	1	21	1	21	culture
	Secondary/persistent infection	\	\	\	\	
	total	1	21	1	21	
[26] Vianna et al., 2007 Oral Microbiology and Immunology	primary endodontic infections	7	24	4	24	culture
	Secondary/persistent infection	\	\	\	\	
	total	7	24	4	24	
[27] Chu et al., 2005 Journal of Endodontics	primary endodontic infections	7	88	2	88	culture
	Secondary/persistent infection	\	\	\	\	
	total	7	88	2	88	
[28] Chavez de Paz et al., 2005 Oral Surgery, Oral Medicine, Oral Pathology, Oral Radiology, and Endodontics	primary endodontic infections	1	100	9	100	PCR
	Secondary/persistent infection	\	\	\	\	
	total	1	100	9	100	
[30] Chavez de Paz et al., 2004 International Endodontic Journal	primary endodontic infections	1	139	18	139	culture
	Secondary/persistent infection	\	\	\	\	
	total	1	139	18	139	
[33] Pinheiro et al., 2003 International Endodontic Journal	primary endodontic infections	\	\	\	\	culture
	Secondary/persistent infection	4	60	1	60	
	total	4	60	1	60	
[35] Sunde et al., 2002 Journal of Endodontics	primary endodontic infections	\	\	\	\	culture
	Secondary/persistent infection	6	36	2	36	
	total	6	36	2	36	
[36] Peters et al., 2002 International Endodontic Journal	primary endodontic infections	5	42	2	42	culture
	Secondary/persistent infection	\	\	\	\	
	total	5	42	2	42	
[38] Sundqvist et al., 1998 Oral Surgery, Oral Medicine, Oral Pathology, Oral Radiology, and Endodontics	primary endodontic infections	\	\	\	\	culture
	Secondary/persistent infection	1	54	1	54	
	total	1	54	1	54	
[41] Sjogren et al., 1997 International Endodontic Journal	primary endodontic infections	2	20	2	20	culture
	Secondary/persistent infection	\	\	\	\	
	total	2	20	2	20	
[44] Debelian et al., 1995 Endodontics & Dental Traumatology	primary endodontic infections	9	26	8	26	culture
	Secondary/persistent infection	\	\	\	\	
	total	9	26	8	26	

**Table 6:** Assessment of risk of bias within the studies (Newcastle–Ottawa scale) with scores 7 to 12 = low quality, 13 to 20 = intermediate quality, and 21 to 24 = high quality.

		Selection			Comparability		Exposure		Score	Outcome
References	Definition of Cases	Representativeness of Cases	Selection of Controls	Definition of Controls	Comparability of Cases and Controls on the Basis of the Design or Analysis	Ascertainment of Exposure	Same Method of Ascertainment for Cases and Controls	Nonresponse Rate		
[13] Pourhajibagher et al., 2018 Photodiagnosis and Photodynamic Therapy	3	3	0	0	0	3	3	0	12	Primary
[14] Grgurevic et al., 2017 Acta Stomatol Croat	3	3	0	0	0	3	3	0	12	Primary
[15] Lysakowska et al., 2016 International Endodontic Journal	3	3	3	3	2	2	3	0	19	Primary, secondary, tertiary
[16] Tennert et al., 2014 Journal of Endodontics	2	3	3	2	2	3	3	0	18	Primary, tertiary
[17] Halbauer et al., 2013 Coll Antropol	1	3	3	1	2	3	3	0	16	Primary, tertiary
[18] Signoretti et al., 2013 Journal of Endodontics	2	1	2	2	2	3	3	0	15	Primary, secondary
[19] Anderson et al., 2013 Journal of Endodontics	3	3	0	0	0	3	3	0	12	Primary
[20] Rocas et al., 2012 Journal of Clinical Microbiology	3	3	0	0	0	3	3	0	12	Primary
[5] Rocas et al., 2011 Journal of Endodontics	2	2	0	0	0	3	3	0	10	Primary
[21] Chugal et al., 2011 Journal of Endodontics	2	2	1	2	2	3	3	0	15	Primary, secondary,
[22] Ledezma-Rasillo et al., 2010 The Journal of Clinical Pediatric Dentistry	3	1	2	2	2	2	3	0	15	Primary, tertiary
[23] Mindere et al., 2010 Stomatologija	2	2	0	0	0	2	2	0	8	Primary
[24] Niazi et al., 2010 Journal of Endodontics	3	1	3	3	2	1	3	0	16	Primary, tertiary
[25] Fujii et al., 2009 Oral Microbiology and Immunology	2	2	0	0	0	2	2	0	8	Primary
[26] Vianna et al., 2007 Oral Microbiology and Immunology	3	2	3	2	2	2	3	0	17	Primary, tertiary
[27] Chu et al., 2005 Journal of Endodontics	3	2	3	3	3	2	3	0	19	Primary, tertiary
[28] Chavez de Paz et al., 2005 Oral Surgery, Oral Medicine, Oral Pathology, Oral Radiology, and Endodontics	3	3	3	3	2	3	3	0	20	Primary, tertiary
[29] Gomes et al., 2004 Oral Microbiology and Immunology	2	2	2	3	3	3	3	0	21	Primary, secondary
[30] Chavez de Paz et al., 2004 International Endodontic Journal	3	3	3	3	3	2	3	0	20	Primary, tertiary
[31] Siqueira et al., 2004 Oral Surgery, Oral Medicine, Oral Pathology, Oral Radiology, and Endodontics	3	1	0	0	0	3	2	0	9	Primary
[32] Hommez et al., 2004 International Endodontic Journal	2	3	2	2	3	3	3	0	18	Primary, secondary
[33] Pinheiro et al., 2003 International Endodontic Journal	2	2	2	2	3	3	3	0	17	Primary, tertiary
[34] Siqueira et al., 2003 Oral Surgery, Oral Medicine, Oral Pathology, Oral Radiology, and Endodontics	2	2	3	2	3	3	3	0	18	Primary, tertiary
[35] Sunde et al., 2002 Journal of Endodontics	2	2	2	2	3	2	3	0	16	Primary, tertiary
[36] Peters et al., 2002 International Endodontic Journal	2	2	3	3	3	2	3	0	18	Primary, tertiary
[37] Rolph et al., 2001 Journal of Clinical Microbiology	3	3	3	3	3	2	3	0	20	Primary, secondary
[38] Sundqvist et al., 1998 Oral Surgery, Oral Medicine, Oral Pathology, Oral Radiology, and Endodontics	2	2	2	2	2	2	3	0	15	Primary, tertiary
[39] Molander et al., 1998 International Endodontic Journal	3	3	0	0	0	2	3	0	11	Primary
[40] Vigil et al., 1997 Journal of Endodontics	3	2	0	0	0	3	2	0	10	Primary
[41] Sjogren et al., 1997 International Endodontic Journal	2	2	2	2	3	2	3	0	16	Primary, tertiary
[42] Gomes et al., 1996 J Dental	3	2	0	0	0	2	2	0	9	Primary
[43] Brauner et al., 1995 International Endodontic Journal	3	2	0	0	0	2	2	0	9	Primary
[44] Debelian et al., 1995 Endodontics & Dental Traumatology	2	2	2	2	3	2	2	0	15	Primary, tertiary
[45] Sundqvist et al., 1992 Oral Microbiology and Immunology	2	2	2	0	0	0	2	0	8	Primary
[46] Fukushima et al., 1990 Journal of Endodontics	2	1	2	0	0	0	2	0	7	Primary
[4] Sundqvist et al., 1989 Journal of Endodontics	3	3	3	0	0	0	2	0	11	Primary
